# Supporting the continuous development and use of a patient partnership framework in European rare disease networks (ERNs): a scoping review of frameworks in the scientific literature

**DOI:** 10.1007/s12687-024-00763-2

**Published:** 2024-12-21

**Authors:** Olivia K. C. Spivack, Mirthe J. Klein Haneveld, Simone Louisse, Graham Slater, Inés Hernando

**Affiliations:** 1 ERNICA, Department of Pediatric Surgery, Erasmus MC Sophia Children’s Hospital, University Medical Centre Rotterdam, Rotterdam, the Netherlands; 2 Amsterdam UMC location University of Amsterdam, Emma Children’s Hospital, Amsterdam, the Netherlands; 3 Amsterdam Reproduction and Development & Amsterdam Public Health Research Institutes, Amsterdam, the Netherlands; 4 ERN-ITHACA, Clinical Genetics Department, Robert Debré University Hospital, Paris, France; 5 EURORDIS - Rare Diseases Europe, Paris, France; 6 ERN GUARD-Heart, Amsterdam UMC location AMC, Amsterdam, the Netherlands; 7 Hart4Onderzoek (Heart4Research), Haarlem, the Netherlands; 8 EAT - Esophageal Atresia Global Support Group, Stuttgart, Germany

**Keywords:** Patient engagement, Involvement, Health system, Orphan disease, Europe

## Abstract

The European Reference Networks (ERNs) for rare and complex diseases offer significant potential for building, maintaining and evaluating patient partnership, for which the recently developed ERN Patient Partnership Framework may serve as guidance. This scoping review aims to identify and describe relevant frameworks published in scientific literature, capturing key learning points to inform future updates of the ERN Patient Partnership Framework and promote its use in practice. MEDLINE, Embase, and the Web of Science Core Collection were searched to identify recently published frameworks (2013–2023) focused on patient partnership and aligned with at least one core ERN activity. Framework characteristics were summarised and information pertaining to their content, structure and practical use was extracted. Twelve relevant frameworks were identified, presenting practical approaches, conceptual understandings or both. Five frameworks focused on areas aligned with specific core ERN activities; others had an overarching scope. Frameworks presented various engagement approaches and employed heterogeneous terminology and development methods. Frameworks differed in their content and structure and presented key considerations for use. Our review underscores the importance of providing clear definitions and explanations of patient partnership. It provides insight into how meaningful, and inclusive patient partnership can be promoted within our diverse ERN context and sheds light on the importance of framework implementation as a prerequisite to structured evaluation. Learning points generated from this review will be used to inform future updates of the ERN Patient Partnership Framework and promote its implementation in practice.

**Supplementary Information:**

The online version contains supplementary material available at 10.1007/s12687-024-00763-2.

## Introduction

European Reference Networks (ERNs) are networks of healthcare providers from across Europe specialised in the care and treatment of rare and complex conditions. Rooted in European legislation (The European Parliament and the Council of the European Union 2011), 24 unique ERNs were established in 2017 by the European Commission, each with their own clinical scope (European Commission 2023). ERNs exist to centralise (disease-specific) expertise from across Europe, pooling together knowledge and resources to improve the quality of care for all patients with rare and complex conditions, regardless of where they live. In this regard, ERNs play a central role in closing knowledge gaps, building capacity and developing novel innovations and solutions for dissemination and implementation. To do so requires structured coordination and evaluation.

In 2015, the European Union Expert Committee on Rare Diseases emphasised the integral role of patient representatives in the ERN system, issuing a recommendation for their involvement in network activities and decision-making processes (European Union Committee of Experts on Rare Diseases 2015). To support the execution of this recommendation, EURORDIS-Rare Disease Europe and the rare disease community established 24 European Patient Advocacy Groups (ePAGs) in 2016, aligned to the clinical scope of each ERN. ePAGs are made up of individual patient representatives (‘ePAG advocates’), who are endorsed by registered patient organisations to represent the voice of their patient community.

Maximising the impact of the ERNs relies on implementation of the ERN system as well as the innovations that it generates. Described as *“the single most important determinant of successful implementation”* (Wensing and Grol 2019), stakeholder involvement is key. With patients and families undeniably key stakeholders in the ERN initiative, ePAG advocates play a crucial role in leveraging the experiential knowledge of these groups. This is particularly significant given the limited scientific understanding surrounding many rare conditions. Fostering a true and meaningful collaboration between ePAG advocates and ERN healthcare professionals is therefore essential.

**Fig. 1:**
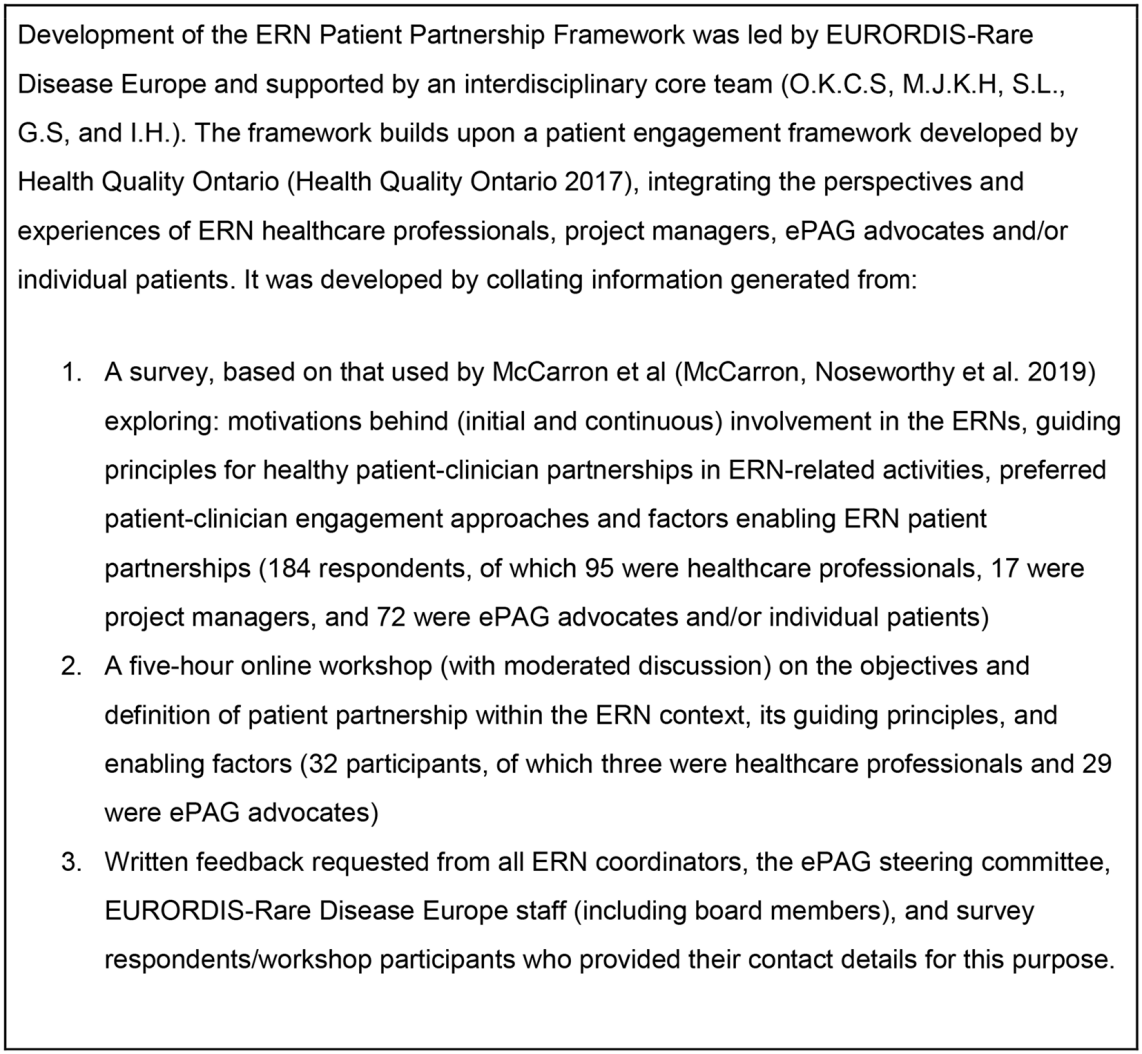
Development process for the ERN Patient Partnership Framework

**Fig. 2:**
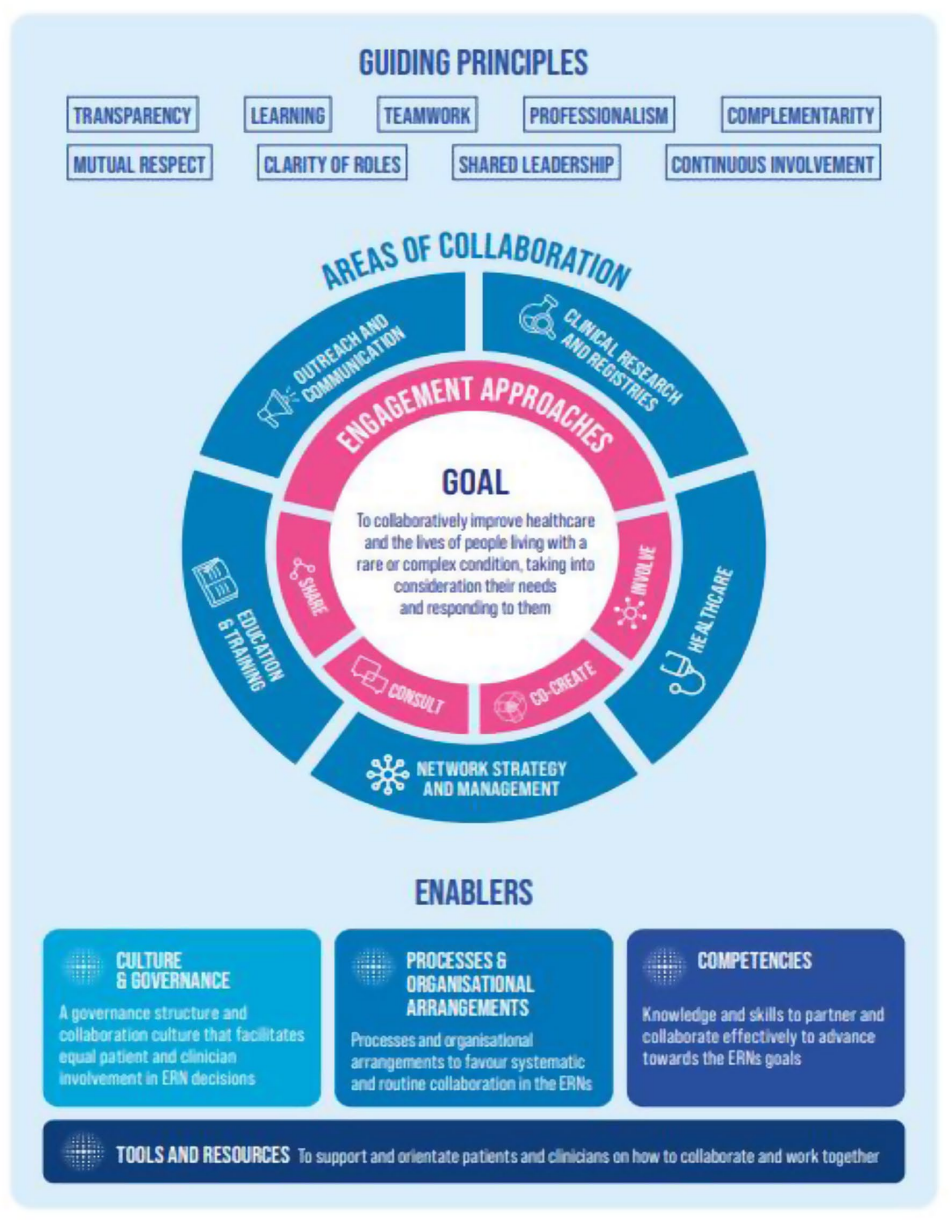
Graphic summary of the ERN Patient Partnership Framework (EURORDIS Rare Disease Europe 2023)

Within the rare disease community, there is growing emphasis on building a culture of patient-clinician partnership, where patient representatives and healthcare professionals are considered equal partners (Rare 2030 Foresight in Rare Disease Policy 2021). This reflects a broader movement towards collaborative partnership approaches in healthcare (Vanstone et al. 2023). In 2023, EURORDIS-Rare Disease Europe led the development of a Patient Partnership Framework for the ERNs (EURORDIS Rare Disease Europe 2023), adopting a similar structure to the patient engagement framework developed by Health Quality Ontario (Health Quality Ontario 2017). Developed with input from ERN healthcare professionals, project managers, and ePAG advocates (see Fig. 1), the ERN Patient Partnership Framework defines key guiding principles and enablers for strong and meaningful patient-clinician partnerships. The framework fosters an ethos of collaboration, giving ePAG advocates an equal seat at the ERN table. However, it recommends selecting and/or combining engagement approaches depending on the specific purpose at hand. Approaches span across a (non-hierarchical) spectrum, ranging from sharing information with patient communities to co-creation (see Fig. 2).

The ERNs offer significant potential for building, maintaining and evaluating patient partnership. While the ERN Patient Partnership Framework has been developed with stakeholder input, valuable insights from frameworks in the scientific literature may be used to promote its continuous improvement. This scoping review aims to identify and describe published frameworks, that are both focused on patient partnership and aligned with at least one of six core ERN activities; (1) Coordination, (2) Dissemination, (3) Evaluation, (4) Registries, data management and analysis (5) Training and education and (6) Clinical practice guidelines and decision-support tools. In doing so, this review aims to capture key learning points to inform future updates of the ERN Patient Partnership Framework and promote its use in practice.

## Methods

### Research team

O.K.C.S and M.J.K.H. are PhD candidates within the European Reference Network for Rare Inherited Congenital Anomalies (ERNICA) and the European Reference Network for Rare Malformation Syndromes, Intellectual and Other Neurodevelopmental Disorders (ERN-ITHACA) respectively. O.K.C.S has five years experience working as an ERN project manager, within ERNICA and the European Reference Network for Rare and/or Complex Craniofacial anomalies, and Ear, Nose, and Throat disorders (ERN CRANIO). She currently works as ERNICA’s implementation coordinator. M.J.K.H has two years experience working in the ERN-ITHACA Guideline Working Group. S.L. and G.S. are ePAG advocates involved with the European Reference Network for Rare and Low Prevalence Complex Diseases of the Heart (ERN GUARD-Heart) and ERNICA respectively, both with long-standing experience working in/with national and international patient groups. S.L. is co-chair of the ePAG steering committee and G.S. sits on the EURORDIS-Rare Diseases Europe Board of Directors. I.H is the ERN and Healthcare Director for EURORDIS-Rare Diseases Europe, with over five years experience working with ERN ePAG advocates.

## Definitions

For the purpose of this review, the term patient partnership was broadly defined, encompassing all approaches involving an interaction between patients and healthcare professionals. In line with the ERN Patient Partnership Framework, patients were defined as all individuals with lived experience of a condition, personally or as a relative or caregiver^1^. Healthcare professionals were defined as individuals providing or supporting the provision of clinical care in healthcare contexts. The ERNs, which are financed through the EU4Health Programme until October 2027 (European Commission 2023), are mandated to carry out a set of activities aligned to seven work packages. Six of these work packages were considered ‘core ERN activities’ for the purpose of this review:

- *Coordination*: Focused on developing the ERN and engaging members and partners.
- *Dissemination*: Focused on dispersing outputs and resources and raising awareness across target groups;
- *Evaluation*: Focused on monitoring and appraising network performance, activities and member contributions;
- *Registries*,* data management and analysis*: Focused on developing and maintaining registries for standardised data collection and analysis;
- *Training and education*: Focused on capacity building and enhancing knowledge and skills;
- *Clinical practice guidelines and decision-support tools*: Focused on developing (and implementing) clinical practice guidelines and other clinical decision-support tools.

### Study design and search strategy

We conducted a scoping review to identify published frameworks that are both focused on patient partnership and aligned with one or more core ERN activity. A scoping review was considered most suitable, given our research question was exploratory in nature. Reporting was guided by the PRISMA Extension for scoping reviews (Tricco et al. 2018). No review protocol was published. A search for relevant articles (published in the English language) was conducted in MEDLINE (Ovid), Embase (embase.com) and Web of Science Core Collection. Search terms were defined by authors (O.K.C.S. and M.J.K.H), with the assistance of an Information Specialist (W.B.). Terms were selected to reflect the heterogeneous terminology used to describe patient partnership in healthcare. A filter was applied to identify recent articles published between 1 January 2013–8 November 2023. See *Supplementary material 1* for the search strings used in each of the databases.

### Framework selection

To identify relevant frameworks, articles underwent initial title/abstract screening followed by full-text review (O.K.C.S and M.J.K.H). During the full-text review stage, snowballing was conducted exclusively for identified review articles meeting the inclusion criteria. Articles were considered eligible if they explicitly self-identified as a ‘framework’ in the title and/or abstract and were focused on ´patient partnership´ in the healthcare context on a topic aligning with one or more core ERN activity. No limitations on disease type or prevalence were imposed to capture insights from a broad range of literature. Table 1 presents the inclusion-exclusion criteria.

**Table 1:** Eligibility criteria

Inclusion	● Full-text peer-reviewed publication in the English language ● Published between 1 January 2013 and 8 November 2023 ● Article explicitly self-identifies as a ‘framework’ in the title/abstract ● Article focuses on guiding patient partnership in the healthcare context on a topic aligning with one or more core ERN activity (1. Coordination 2. Dissemination 3. Evaluation 4. Registries, data management and analysis 5. Training and education 6. Clinical practice guidelines and clinical decision-support tools) ● Article describes the development process of said framework ● Article describes a framework which focuses on the interaction between two groups, specifically including patients and healthcare professionals
Exclusion	● Framework centres on partnering with patients in individual patient care and/or in other activities outside the scope of this review (e.g. in research, medicine development, health technology assessment) ● Framework focuses exclusively on public engagement (not including patients and health care professionals) ● Framework focuses solely on describing, defining and/or evaluating patient partnership ● Not successful in retrieving full-text publication

### Data charting and synthesis

The following information was extracted from articles meeting the inclusion criteria; author(s), year of publication, countr(ies) of development and/or leading organisation (if relevant), purpose and context of the framework, topic of the framework (aligned with pre-defined core ERN activities), type of framework (conceptual versus practical), framework development process, target populations, framework content and structure, and self-reported considerations for use. Data was charted in duplicate by two authors (O.S and M.K), tabulated, and reviewed by all authors. No critical appraisal of the frameworks´ quality was conducted considering the exploratory nature of this review and framework heterogeneity.

## Results

**Fig. 3:**
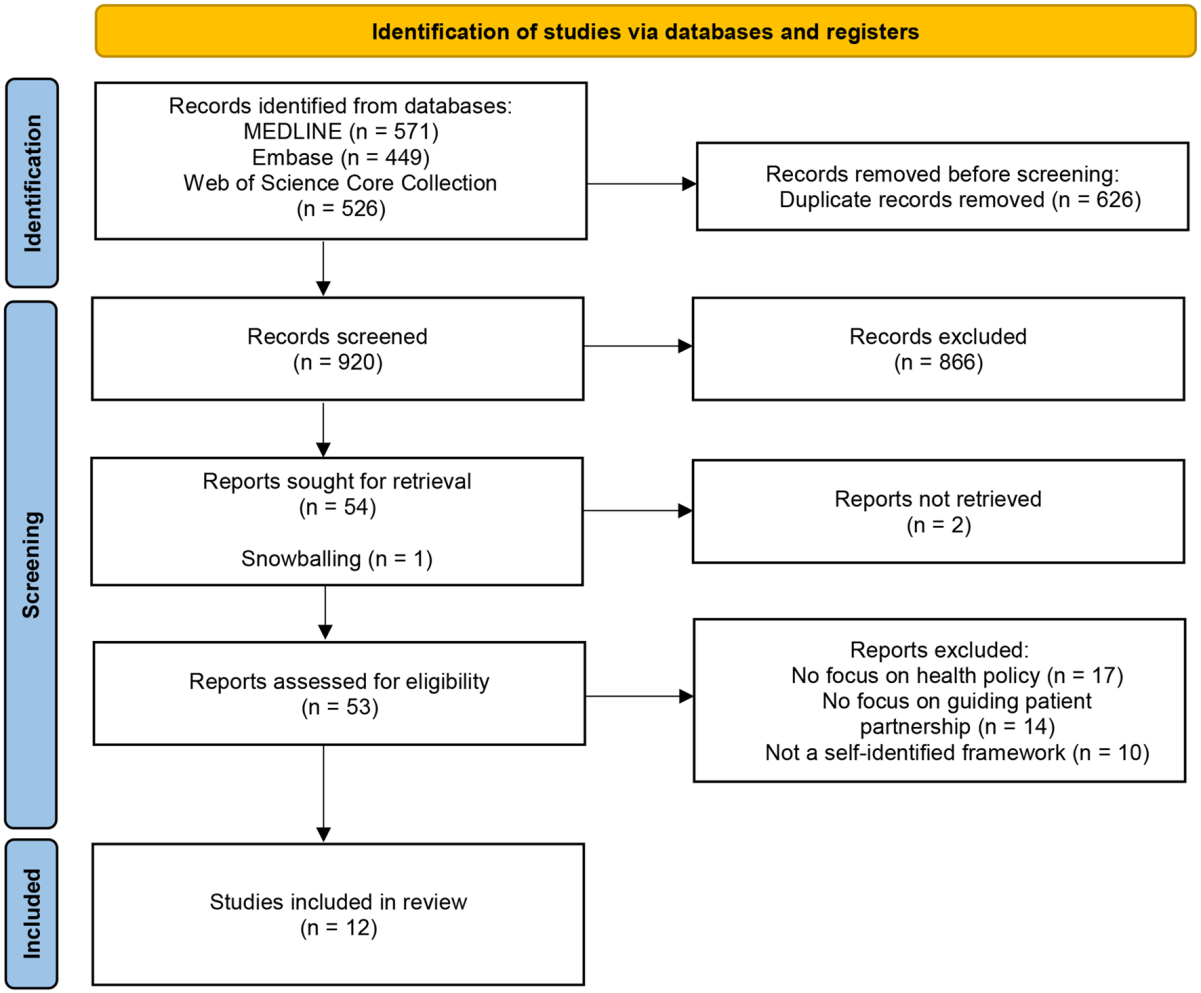
PRISMA Flow Diagram

Our initial search yielded 921 records after duplicate removal, from which 12 frameworks met the inclusion criteria. The article selection process is visualised in a PRISMA flow diagram (Page et al. 2021) (see Fig. 3). Framework characteristics are presented in Table 2.

Five frameworks focused on specific core ERN activities including Clinical practice guidelines and decision-support tools (*n* = 2); Registries, data management and analysis (*n* = 1); Coordination (*n* = 1); and Training and education (*n* = 1) and seven had an overarching scope. Frameworks presented practical approaches in the form of step-by-step guidance (*n* = 3), conceptual understandings in the form of guiding principles, capabilities, or key insights (*n* = 6), or a combination of both (*n* = 3).

Framework development processes were heterogeneous. Literature and document reviews, key informant interviews, surveys, workshops, Delphi consensus methods, existing frameworks, and/or real-life author experiences were used to inform development. Ten frameworks were developed in the United States of America, Canada, or Australia and one was developed in the United Kingdom. One framework was produced by a European scientific association.

The frameworks refer to various approaches involving an interaction between patients and healthcare professionals. Heterogeneous terminology is used, such as patient and/or family engagement, patient and/or public involvement, and (patient and staff) partnership. Framework content and structure as well as authors’ self-reported considerations for use are described in Table 3.

**Table 2:** Characteristics of included frameworks organised by the ‘core ERN activities’ selected for this study

Author and year	Countr(ies) of development and/or leading organisation (if relevant)	Purpose and context of the framework	Type of framework	Framework development process	Target populations
**Overarching**					
**(**Carman et al. 2013)	United States of America	To propose a model of patient engagement relevant to the healthcare system	Conceptual	By authors (through experience) in conjunction with patient and family representatives	Clinicians, healthcare organisations, researchers, and policy makers; Patients, families, caregivers, and other consumers and citizens.
**(**Cox et al. 2022)	Australia	To outline the capabilities required for successful patient and staff partnerships in quality improvement at the health service level and provide recommendations for building and supporting these capabilities	Conceptual	Scoping review co-produced by patients and health professionals	Healthcare staff; Health service consumers, their families and carers, and members of the public.
**(**Dworetzky et al. 2023)	United States of America	To support meaningful family engagement in health system-level activities	Practical and conceptual	Engagement of an expert family/professional workgroup whose members represented key constituencies and diverse geography, race/ethnicity and areas of expertise; Review of peer-reviewed publications and grey literature; Series of key informant interviews (with family advocates and professionals) to identify best practices	Professionals and families in child and family-serving health organisations
**(**Macklin et al. 2019)	Canada; University Health Network for patients and families who have received care for heart failure, heart transplant, or mechanical circulatory support (left ventricular assist device)	To engage patients and caregivers in Care Delivery and Policy, Patient Advocacy, Peer Support, and Research at the University Health Network	Practical and conceptual	Adaptation from existing documents; Strategy for Patient-Oriented Research (Strategy for Patient-Oriented Research (SPOR) 2014) and Health Quality Ontario’s framework (Health Quality Ontario 2017)	Clinicians providing care for heart failure, heart transplant, or mechanical circulatory support (left ventricular assist device) (at the University Health Network and other heart failure and transplant institutions across Canada); Patients and families who have received care for heart failure, heart transplant, or mechanical circulatory support (left ventricular assist device) (at the University Health Network and other heart failure and transplant institutions across Canada)
**(**McCarron et al. 2020)	Canada	To support and sustain patient and family engagement in healthcare decision making through knowledge of patient and family motivations	Conceptual	A regional survey (with patients and families), informed by a scoping review and interviews with patient and family members, and four regional workshops (with professionals and patient and family members)	Professionals working within the Alberta health system; Patient and family members involved in the Alberta health system
**(**Restall 2015**)**	Canada	To guide the development and evaluation of citizen-user involvement in mental health policymaking	Conceptual	Qualitative instrumental case study involving interviews with informants from four policy actor groups (citizen-users, representatives of advocacy organisations, government officials [elected representatives and bureaucrats] and service providers) and a review of policy documents	People who use services (citizen-users) and other policy actors
**(**Wong-Rieger 2017**).**	Canada	To understand patient participation, grounded in real experiences of actively engaged patients, with reference to their political, social, economic and healthcare context	Conceptual	Review of several theoretical models and real-life experiences of grassroots patient organisations across a variety of diseases, countries and contexts	People working within health systems; Patient communities/organisations
**Coordination**					
**(**Duncanson et al. 2020)	Australia; Better Evidence and Translation in Chronic Kidney Disease (BEAT-CKD) research collaboration	To partner with consumers in organising scientific meetings in nephrology	Practical	Author experiences from organisation of consumer research sessions	Researchers/health professionals in nephrology; Consumers (patients, carers and family members) affected by kidney disease, including people with chronic kidney disease, people receiving peritoneal dialysis, hemodialysis, carers/family members of dialysis patients, and transplant recipients
**Registries**,** data management and analysis**					
**(**Carlton et al. 2020)	United Kingdom	To serve as a prompt and reference point for public engagement in the development, dissemination and promotion of Patient-Reported Outcome Measures (PROMs)	Practical	Author experiences based upon experiences as researchers working with public representatives in PROM development	PROM developers; Public includes patients, potential patients, carers and people who use health and social care services, as well as organisations that represent people who use these services
**Training and education**					
**(**Price et al. 2021)	United States of America	To strengthen commons (the cultural and natural resources accessible to all members of society) and co-production principles for medical education	Conceptual	Author experiences of commons and co-production principles in medical education	Healthcare professionals involved in medical education; Patients and public
**Clinical practice guidelines and decision-support tools**					
**(**Armstrong et al. 2017)	United States of America	To provide an overview of the steps and options for patient engagement across the clinical practice guideline lifecycle at the developer/committee and individual guideline project level	Practical	Review of published literature and pragmatic (author and external) experiences	Individuals involved in clinical practice guideline panels; Patients as “all lay stakeholders filling this role”
**(**Björkqvist et al. 2021)	European Association of Urology	To generate meaningful patient involvement in clinical practice guideline production and implementation in the context of genitourinary cancers	Practical and Conceptual	With integrated outputs from a systematic review of patient involvement methodologies, interviews with key stakeholders (involving an assessment of barriers and enablers), and the results of a Delphi process involving patients and clinicians	Clinicians caring for genitourinary cancers, including urologists, nurses, and medical and clinical oncologists; Patients with genitourinary cancers (prostate, renal, bladder, or testicular cancer)

**Table 3:** Framework content and structure and self-reported considerations for use, organised by the ‘core ERN activities’ selected for this study

Author, year and type of framework	Framework content and structure	Self-reported considerations for use
Overarching		
**(**Carman et al. 2013) Conceptual	Multi-dimensional framework describing three levels of engagement (direct care, organisational design and governance, and policy making), a three-stage continuum of engagement (consultation, involvement, partnership and shared leadership, whereby the approach is defined with the patient) and three sets of factors influencing engagement (pertaining to the patient, the organisation and the society).	● Attention should be paid to organisational and societal barriers to patient engagement ● Monitoring progress on patient engagement requires the use of robust measures; these results should inform subsequent engagement efforts
**(**Cox et al. 2022) Conceptual	Ten capabilities, including knowledge, skills and attitudes required for both staff and patients, in three domains (personal attributes, relationships and communication, and philosophies, models, and practices) and across the domains (sharing power and leadership).	● The framework could be used to support individual learning plans, gap analyses of organisational learning resources and training needs analyses ● Team co-learning, rather than learning in silos, is recommended; consideration should be given to learning and development approaches
**(**Dworetzky et al. 2023) Practical and conceptual	Four action-oriented domains of family engagement in systems-level initiatives, each with a definition and key criteria: (1) Commitment (2) Transparency (3) Representation (4) Impact.	● The Family Engagement in Systems Assessment Tool (FESAT) (Family Voices 2022) may be used to plan, assess, and improve family engagement in systems-level activities over time.
**(**Macklin et al. 2019) Practical and conceptual	Four levels of patient engagement (Share, Consult, Deliberate, Collaborate) to engage patients in four priority areas (Care delivery and policy, Patient advocacy, Peer support work, and Research), adapted from the Strategy for Patient-Oriented Research (Strategy for Patient-Oriented Research (SPOR) 2014) and Health Quality Ontario’s framework (Health Quality Ontario 2017).	Possible solutions are provided to overcome the following barriers to patient engagement: ● Representation, ● Training/resources ● Tokenism ● Compensation ● Maintaining patient/caregiver participation ● Evaluation, e.g. using the McMaster’s Public and Patient Engagement Tool (PPEET) (Abelson et al. 2016).
**(**McCarron et al. 2020) Conceptual	Seven motivations (Self-fulfillment, Improving healthcare, Compensation, Influence, Learning new things, Conditional, and Perks) and 24 statements arranged by three phases of engagement: Why I got involved; Why I continue to be involved; and What I need to strengthen my involvement.	● It is equally important to focus efforts on retaining and sustaining patient and family involvement (as it is attracting patient involvement) ● Choice of engagement opportunity enables patient and family members to maximise the value they receive
**(**Restall 2015**)** Conceptual	Four outcome dimensions (Personal, Substantive, Instrumental and Normative) organised into the micro-, meso- and macro-level, with key questions for development and evaluation of citizen-user involvement at each dimension.	● Involvement processes should begin with a clear understanding of the outcome targets ● Positive macro, meso and micro level outcomes should be pursued ● There should be awareness of both the potential risks and rewards of involvement ● Adequate resources for involvement mechanisms should be ensured
**(**Wong-Rieger 2017**)** Conceptual	Four-mode framework of the transition from patient advocacy to partnership, defined by one axis as individual vs. collective action and the other axis as activities ‘outside’ vs. ‘inside’ the system. The four quadrants are labeled as Advocacy, Activism, Reform and Broker, and engagement is further refined by whether the participation is ‘pushed’ by the group or ‘pulled’ by the system.	● The impact of culture, opportunity and preferences of patient groups should be recognised ● Education about how the system works and opportunities for skills training and engagement needs to be provided
**Coordination**		
**(**Duncanson et al. 2020) Practical	Eight key strategies for partnering with consumers in scientific meetings in nephrology.	● Outcomes and follow-on actions from the partnership are to be fed back to consumers to bring the activity full circle ● Accessibility for consumers (e.g. timing, digital participation, cost compensation) should be considered
**Registries**,** data management and analysis**		
**(**Carlton et al. 2020) Practical	Public involvement strategies for the 11 stages of PROM development, dissemination and promotion- providing guidance and not prescriptive rules.	Consideration should be given to several factors when developing collaborative involvement initiatives related to PROM development: ● Need for a clear public involvement (PI) plan ● Meeting and involvement practicalities ● Preparation and delivery of PI tasks ● Building and sustaining relationships ● Ethical implications, wellbeing of PI partners and appropriate commitment ● Reporting of PI activities ● Developing instruments for children or those with limited capacity ● Developing instruments for very rare conditions ● The use of PI in international PROM development ● Developing instruments for use in preference-based measures ● Ensuring optimum PI within PRO development
**Training and education**		
**(**Price et al. 2021) Conceptual	The principle of Everyone Included, “where everyone is trusted and respected for the expertise they bring, where openness and experimentation is the norm, people have personal ownership of health, individual stories have a global impact, and the patient voice and choice is a part of all stakeholder decisions”, illustrated with practice examples from medical education.	● Points for attention include the importance of building trust (e.g. through storytelling), early involvement, co-creation (e.g. through co-producing resources) and working with marginalised groups and intergenerational participants
**Clinical practice guidelines and decision-support tools**		
**(**Armstrong et al. 2017) Practical	Patient engagement can be organised throughout the guideline development process, for which various strategies can be applied depending on the goals of engagement. The purpose and proposed methods of patient engagement are provided for 10 steps involved in the clinical practice guideline lifecycle.	● Factors such as resource availability, time sensitivity, and disease-specific considerations may influence when and how guideline panels engage patients ● Combining multiple engagement strategies may be beneficial ● Training for both patients and the panels engaging them may be needed ● Mechanisms to evaluate whether patient engagement was meaningful (involving patients) should be applied
**(**Björkqvist et al. 2021) Practical and conceptual	Three domains for meaningful patient involvement: (1) Commitment to involving patients at a strategic level; (2) Acceptance of patient involvement as part of the organisational culture; (3) Patient engagement and involvement in relevant stages of the guideline development process.	● The EVOLVE framework is undergoing ongoing evaluation and adaptation within the European Association of Urology; an evaluation strategy for assessing the impact of patient involvement is to be developed.

## Discussion

This scoping review identified 12 frameworks on patient partnership aligned to core ERN activities. Developed predominantly in English-speaking countries, frameworks differed in terms of their topic area and scope and employed heterogeneous terminology and development methods. While their content and structure vary, all frameworks provide a number of considerations for practical application. Below, we outline four key learning points captured by this review, to inform future updates of the ERN Patient Partnership Framework and promote its use in practice. See Table 4 for an overview of key learning points captured by this review and implications for further development and use of the ERN Patient Partnership Framework.

### Use and promote the definition of ERN Patient Partnership: Patients and healthcare professionals as equal partners in the selection of appropriate engagement approaches

The identified frameworks employ heterogeneous terminology for the collaboration between patients and healthcare professionals. This highlights the importance of having a clear definition of patient partnership and an explanation of its goals, within the ERN Patient Partnership Framework itself and for the purpose of its dissemination and evaluation. The ERN Patient Partnership Framework defines patient partnership as *“a mutual relationship between patients and health professionals*,* where input from people living with a rare disease or caring for someone with a rare disease routinely and formally informs the Networks’ collaborative activities and decision-making [which] implies considering health professionals and patients involved in the Networks as equal partners in all ERN activities and domains”* (EURORDIS Rare Disease Europe 2023). This definition originated from the Rare 2030 foresight study (Rare 2030 Foresight in Rare Disease Policy 2021). It was subsequently adapted to the ERN context and agreed upon as part of the 5-hour framework development workshop involving key ERN stakeholders.

This definition reflects the importance of working as equal partners within a team, requiring a strong foundation of shared power and leadership and team co-learning described by Cox et al. (Cox et al. 2022), as well as formal structures in which patient representatives can work as ‘brokers’ within the ERN system, described by Wong-Rieger (Wong-Rieger 2017). Furthermore, in line with the ERN Patient Partnership Framework, frameworks present patient engagement approaches along a (non-hierarchical) continuum spanning from sharing to co-creation (Carman et al. 2013; Macklin et al. 2019). Rather than advocating for an identical and equal distribution of tasks, the ERN Patient Partnership Framework promotes the collaborative selection of engagement approaches. To work in this way, roles and responsibilities must be clear to all involved. Defining clear roles and responsibilities is specifically recognised in the ERN Patient Partnership Framework as one of the nine guiding principles promoting healthy, meaningful patient partnerships.

### Strive for meaningful patient partnership that is outcome and/or value based

Several frameworks identified in this review underscore the importance of meaningful patient engagement which is outcome-driven and/or value-based. Macklin et al. (Macklin et al. 2019) identify tokenism as a key barrier to effective patient engagement and propose several solutions to mitigate it. Such solutions align with guidance provided in the ERN Patient Partnership Framework, emphasising the importance of advance discussion and communication on patient engagement approaches, roles and responsibilities, as well as the celebration of good results and progress.

Restall et al. (Restall 2015) advocate for an upfront, clear understanding of the desired and potential outcomes of patient involvement, while McCarron et al. (McCarron et al. 2020) emphasise the importance of understanding patient and family motivations to create meaningful engagement opportunities. The ERN Patient Partnership Framework outlines a set of guiding principles for meaningful collaboration, co-identified by patients and healthcare professionals: transparency, learning, teamwork, professionalism, complementarity, mutual respect, clarity of roles and responsibilities, shared leadership, and continuous involvement (EURORDIS Rare Disease Europe 2023). When selecting appropriate engagement approaches, the framework also recognises the importance of identifying desired outcomes.

Patient-clinician motivations for both initial and continuous ERN involvement were assessed as part of the framework’s development process. Specifically, these were captured in the survey circulated to stakeholders, which was based on a survey previously administered by McCarron et al. in 2019 (McCarron et al. 2019). Alongside other inputs, results of McCarron et al.’s survey (McCarron et al. 2019) were used to inform development of the aforementioned framework developed by McCarron et al. in 2020 (McCarron et al. 2020).

However, while ERN patient-clinician motivations have been assessed, their consideration during the prioritisation of collaborative activities and selection of engagement approaches has not been explicitly encouraged in the ERN Patient Partnership Framework. Such encouragement may help to enhance the value of patient-clinician partnerships within the ERN context.

### Consider diversity and context to ensure patient partnership is both meaningful and inclusive

A number of frameworks identified by this review suggest that the type and nature of patient engagement may depend on several factors relevant to the individuals involved, such as their characteristics (culture, age/generation, condition), experiences, opportunities and preferences (Armstrong et al. 2017; Wong-Rieger 2017; Carlton et al. 2020; Duncanson et al. 2020; McCarron et al. 2020; Price et al. 2021). While such factors are not currently acknowledged in the ERN Patient Partnership Framework, their thoughtful consideration may be of added value in the pursuit of engagement that is both meaningful and inclusive. This is particularly significant given the diverse, European context in which the ERNs operate. Remaining mindful of accessibility needs within our target group is paramount and specific attention pertaining to the timing, nature and cost of participation is warranted (Duncanson et al. 2020). With there being 24 official languages within the European Union (EU) (European Union), language barriers/needs are also a crucial consideration for our ERN context. Rather than limiting participation to English-speaking individuals, the discussion of language barriers/needs may be integrated into the process of collaboratively selecting (appropriate) engagement approaches. This can be supported by translation services or tools, as required.

While fostering inclusivity may be key to recruiting and retaining ePAG advocates with varied backgrounds and experiences, it is important to recognise their position in representing patient communities. ePAG advocates play a crucial role in understanding their patient community, assessing and communicating the suitability of patient engagement approaches, and recognising their own limits and capabilities. As already recognised in the ERN Patient Partnership Framework, patient representatives “*play a fundamental role in connecting the networks with the wider rare disease patient community and*,* where relevant*,* championing the diversity of views*” (EURORDIS Rare Disease Europe 2023). It also acknowledges that they require support *“to capture and analyse the voice of their communities”*. This demands a level of commitment, time and resources (Restall 2015) from the ERN community as a whole.

### Foster a culture of continuous improvement by actively addressing framework evaluation and implementation

A number of frameworks identified note the importance of evaluating the experience and/or impact of patient engagement (Carman et al. 2013; Armstrong et al. 2017; Macklin et al. 2019; Björkqvist et al. 2021; Dworetzky et al. 2023) and feeding back outcomes to inform subsequent engagement efforts (Carman et al. 2013; Duncanson et al. 2020). The ERN Patient Partnership Framework also advocates for continuous improvement backed by a rigorous evaluation of impact and experience (Abelson et al. 2023). Some additional impact measurement tools were identified in our review (Family Voices 2022). Efforts are underway within EURORDIS to test impact measurement tools for use within the ERN context.

The value of evaluation, however, relies on its effective implementation in practice. It is of note that a number of identified frameworks recognise various barriers to effective patient engagement on an individual, organisational and/or societal level (Carman et al. 2013; Armstrong et al. 2017; Macklin et al. 2019; Duncanson et al. 2020; Björkqvist et al. 2021). Barriers were also considered during development of the ERN Patient Partnership Framework and used to inform the development of recommendations, referred to as ‘enablers’. To operationalise such recommendations, the framework encourages the use of practical, task-specific guides (e.g. EURORDIS’ practical guide for partnership in guideline development), which can be likened to the ‘practical frameworks’ identified in this review. However, such frameworks also have recognised barriers (Armstrong et al. 2017). Furthermore, it is to be acknowledged that ERNs may endeavour to endorse relevant resources (e.g. clinical guidelines) developed by scientific or professional societies. Such societies may experience a unique set of barriers to patient partnership.

Given the pivotal role of stakeholder involvement in maximising the impact of the ERNs, active efforts are needed to overcome barriers and increase (sustained) uptake of both the ERN Patient Partnership Framework and the practical guides it recommends. For some ERNs, this may involve initiating a dialogue with relevant scientific and professional societies overlapping in focus. The field of implementation science, the scientific study of methods to increase uptake of evidence-based practices (Nilsen 2015), can offer valuable insights to promote successful implementation. Exploring and evaluating implementation success will also advance knowledge on how to effectively engage patients as stakeholders in (ERN) implementation initiatives.

**Table 4:** Key learning points captured by this review and implications for further development and use of the ERN Patient Partnership Framework

Key learning point	Implications for further development and/or use of the ERN Patient Partnership Framework:
**1. Use and promote the definition of ERN patient partnership: Patients and healthcare professionals as equal partners in the selection of appropriate engagement approaches**	- Foster a shared understanding of patient partnership across all stakeholders – by consistently using the definition of patient partnership included in the ERN Patient Partnership Framework (e.g. for the purposes of dissemination and evaluation)^*^ - Promote collaborative decision-making for the selection of engagement approaches - Agree on clear roles and responsibilities for all involved stakeholders
**2. Strive for meaningful patient partnership that is outcome and/or value based**	- Align with guidance provided in the ERN Patient Partnership Framework, such as the guiding principles for meaningful patient partnership – transparency, learning, teamwork, professionalism, complementarity, mutual respect, clarity of roles and responsibilities, shared leadership and continuous involvement - Identify desired outcomes when selecting appropriate engagement approaches - *Consider stakeholder motivations when prioritising collaborative activities and selecting appropriate engagement approaches*
3. **Consider diversity and context to ensure patient partnership is both meaningful and inclusive**	- *Consider factors relevant to the individuals involved*,* such as their characteristics (culture*,* age/generation*,* condition)*,* experiences*,* opportunities and preferences when selecting appropriate engagement approaches* - Establish a support structure for ERN patient representatives to capture and analyse the voice of their patient communities
4. **Foster a culture of continuous improvement by actively addressing framework evaluation and implementation**	- Adopt a structured approach to framework evaluation - *Adopt an active*,* structured approach to framework implementation*,* as a prerequisite to evaluation*

*Points written in Italic font are not yet (explicitly) included or encouraged in the ERN Patient Partnership Framework and will be considered for inclusion in future updates

### Limitations

There are several limitations to our study. The search strategy, despite having been developed with the support of an experienced information specialist, may not have identified all articles relevant to this review. Articles may have been missed due to heterogeneous terminology used in scientific literature for patient engagement and for individuals with lived experience; for example, relevant articles using the term ´public´ may not have been captured. Furthermore, we may have overlooked content-relevant articles not explicitly identifying as a framework.

The frameworks identified in this review were predominantly developed in native English-speaking countries. This is likely explained by the inclusion of English language articles only. It is also of note that no framework identified was specifically focused on patient partnership in the rare disease context. Our review imposed no restrictions on disease type or prevalence. However, careful consideration was given to the application of key insights within the ERN context.

This review does not attempt to capture articles pertaining to all ERN activities e.g. with those relevant to the ERN work package on digital health not included. For many ERNs, efforts in the field of digital health are limited to use of the Clinical Patient Management System (CPMS), an ERN-specific virtual consultation platform. Many ERNs are also facilitating scientific research. However, having already received extensive research attention, patient partnership in scientific research was considered a field in its own right.

No quality assessment was applied to any of the frameworks selected, nor did we come across any evaluations of the identified frameworks during the article selection process. Evidence is lacking on the impact of varying and multifaceted patient engagement approaches (Carman et al. 2013; Armstrong et al. 2017; Cox et al. 2022). The identification of key learning points relied strongly on author interpretation based on previous experience.

## Conclusion

Our review highlights the importance of providing clear definitions and explanations of ERN patient partnership, within the framework itself and for the purpose of its dissemination and evaluation. It provides insight into how meaningful, and inclusive patient partnership can be promoted within our diverse ERN context. Furthermore, in fostering a culture of continuous improvement, this review shines a light on framework implementation as a prerequisite to structured evaluation. Learning points generated from this review will be used to inform future updates of the ERN Patient Partnership Framework and promote its implementation in practice.

## Electronic Supplementary Material

Below is the link to the electronic supplementary material.

Supplementary Material 1

## Data Availability

No datasets were generated or analysed during the current study.

## Footnotes

1 For the purpose of the ERN Patient Partnership Framework, the term ‘patient’ was used to refer to individuals living with a rare disease and their family members who participate in the ERNs. This terminology was employed to enhance the framework’s clarity and should not be misconstrued as describing individuals who are passively the subject of a rare or complex condition.
