# Supplementary material for: Supporting the continuous development and use of a patient partnership framework in European rare disease networks (ERNs): a scoping review of frameworks in the scientific literature
Source: J Community Genet. 2024 Dec 21;16(1):15–28. doi: 10.1007/s12687-024-00763-2 (PMC11950562; doi:10.1007/s12687-024-00763-2)
Supplement: Supplementary file 1 — Supplementary Material 1 [file 12687_2024_763_MOESM1_ESM.pdf]

Supplementary material 1 for:

Supporting the continuous development and use of a patient partnership framework in European rare disease networks (ERNs): a scoping review of frameworks in the scientific literature

Journal of Community Genetics

Olivia K. C. Spivack*, Mirthe J. Klein Haneveld*, Simone Louisse, Graham Slater, Inés Hernando
**Joint first authorship. Both authors contributed equally.*

Corresponding author: O.K.C. Spivack, Department of Pediatric Surgery / ERNICA coordination office, Erasmus MC Sophia Children's Hospital, University Medical Centre Rotterdam, Rotterdam, Netherlands.
o.spivack@erasmusmc.nl

Search strings per database:

Medline (Ovid)	(* Patient Participation / OR (((Patient* OR client* OR family OR parent* OR informal*-caregiver* OR user OR stakeholder*) ADJ3 (Engagement* OR involvement* OR partnership* OR collaborat* OR cooperat* OR co-creat* OR contribut* OR participat* OR representat* OR alliance* OR empowerment*))).ti.) AND (((framework* OR frame-work* OR guidance* OR princip*) ADJ6 (evaluat* OR novel* OR new OR implement* OR develop*)).ab. OR (framework* OR frame-work* OR guidance* OR princip*).ti.) AND english.la.
Embase (Embase.com)	('patient engagement'/mj OR 'patient participation'/mj OR (((Patient* OR client* OR family OR parent* OR informal*-caregiver* OR user OR stakeholder*) NEAR/3 (Engagement* OR involvement* OR partnership* OR collaborat* OR cooperat* OR co-creat* OR contribut* OR participat* OR representat* OR alliance* OR empowerment*))):ti) AND (((framework* OR frame-work* OR guidance* OR princip*) NEAR/6 (evaluat* OR novel* OR new OR implement* OR develop*)):ab OR (framework* OR frame-work* OR guidance* OR princip*):ti) NOT [conference abstract]/lim AND [english]/lim
Web of Science Core Collection	TI=((((Patient* OR client* OR family OR parent* OR informal*-caregiver* OR user OR stakeholder*) NEAR/2 (Engagement* OR involvement* OR partnership* OR collaborat* OR cooperat* OR co-creat* OR contribut* OR participat* OR representat* OR alliance* OR empowerment*)))) AND (TS=((framework* OR frame-work* OR guidance* OR princip*) NEAR/5 (evaluat* OR novel* OR new OR implement* OR develop*)) OR TI=(framework* OR frame-work* OR guidance* OR princip*)) AND DT=(article) AND LA=(english)
